# CNN-Siam: multimodal siamese CNN-based deep learning approach for drug‒drug interaction prediction

**DOI:** 10.1186/s12859-023-05242-y

**Published:** 2023-03-23

**Authors:** Zihao Yang, Kuiyuan Tong, Shiyu Jin, Shiyan Wang, Chao Yang, Feng Jiang

**Affiliations:** 1 Faculty of Life Science and Food Engineering, Huaiyin Institute of Technology, Huaian, 223003 Jiangsu China; 2 Translational Institute for Cancer Pain, Chongming Hospital Affiliated to Shanghai University of Health & Medicine Sciences (Xinhua Hospital Chongming Branch), Shanghai, 202150 China; 3 Jiangsu Provincial Engineering Laboratory for Biomass Conversion and Process Integration, Huaiyin Institute of Technology, Huaian, 223003 Jiangsu China

**Keywords:** Drug‒drug interaction, Deep learning, Siamese network, Convolutional neural network, Multimodal

## Abstract

**Background:**

Drug‒drug interactions (DDIs) are reactions between two or more drugs, i.e., possible situations that occur when two or more drugs are used simultaneously. DDIs act as an important link in both drug development and clinical treatment. Since it is not possible to study the interactions of such a large number of drugs using experimental means, a computer-based deep learning solution is always worth investigating. We propose a deep learning-based model that uses twin convolutional neural networks to learn representations from multimodal drug data and to make predictions about the possible types of drug effects.

**Results:**

In this paper, we propose a novel convolutional neural network algorithm using a Siamese network architecture called CNN-Siam. CNN-Siam uses a convolutional neural network (CNN) as a backbone network in the form of a twin network architecture to learn the feature representation of drug pairs from multimodal data of drugs (including chemical substructures, targets and enzymes). Moreover, this network is used to predict the types of drug interactions with the best optimization algorithms available (RAdam and LookAhead). The experimental data show that the CNN-Siam achieves an area under the precision-recall (AUPR) curve score of 0.96 on the benchmark dataset and a correct rate of 92%. These results are significant improvements compared to the state-of-the-art method (from 86 to 92%) and demonstrate the robustness of the CNN-Siam and the superiority of the new optimization algorithm through ablation experiments.

**Conclusion:**

The experimental results show that our multimodal siamese convolutional neural network can accurately predict DDIs, and the Siamese network architecture is able to learn the feature representation of drug pairs better than individual networks. CNN-Siam outperforms other state-of-the-art algorithms with the combination of data enhancement and better optimizers. But at the same time, CNN-Siam has some drawbacks, longer training time, generalization needs to be improved, and poorer classification results on some classes.

## Background

Drug‒drug interactions (DDIs) are reactions that occur between two or more drugs. When two or more drugs are used together, the following three types of drug‒drug interactions may occur: co-interaction, antagonism, and no reaction. All these reactions can affect the therapeutic effects of drugs. Therefore, DDIs play an important role in both drug development and clinical treatment sessions. The prediction of DDIs is a very complex task because drug interactions are determined by a combination of factors, including the structure, function, and biological activity of the drug. Since experiments cannot be used to individually study the interactions of such a large number of drug classes, technical solutions based on deep computer learning are always worth exploring.

With the rapid increase in the computing power of computer hardware in the last decade, the branch of machine learning that focuses on neural network models, such as deep learning, has developed rapidly. The emergence of AlexNet [1] initially displayed the power of deep neural network models. In recent years, methods using deep learning algorithms that predict DDIs have proliferated and have surpassed traditional machine learning model-based methods in terms of the performance. For example, DeepDDI [2] uses the structural information of drug pairs as the input to deep neural networks to predict 86 types of important DDIs. Liu et al. in [3] used a CNN to predict DDIs with good results. Some subsequent work using multimodal information has appeared. Lee [4] et al. used a deep autoencoder model to learn structural similarity profiles, gene ontology term similarity profiles, and target gene similarity profiles of drug pairs to predict DDIs. AttentionDDI [5] uses the drug target, pathway, and gene expression profile data for the dichotomous task of predicting whether a drug will have an effect or not. One of the better methods used for multimodal information is DDIMDL [6], which uses multimodal data to predict the types of drug interactions. They extracted the chemical structures, targets, enzymes, and pathway information for 572 drugs, 74,528 drug pairs from the DrugBank [7] online database of drugs, counted the more common 65 drug interaction types, and then used a multilayer perceptron (MLP) as the model architecture. Their approach was to input the single modality information of each drug pair into the model, obtain the corresponding prediction output, and finally average the output obtained from the four modalities as the final prediction, which achieved good results. All the above multimodal related works have achieved more satisfactory results. From above and recent reports [8–11] we can conclude that the multimodal direction is an important area for future research on DDI prediction methods.

In the open-source implementation of DDIMDL, we found that some improvements could be made by inputting the four modal data into the same model instead of four separate models. This process does not have a parallel effect, and its efficiency is greatly reduced. Moreover, the model architecture of DDIMDL is simpler and cannot fully exploit the information of the multimodal data. Some subsequent works improved the model architecture of DDIMDL, and their proposed algorithm called CNN-DDI [12] replaced the original multilayer perceptron of convolutional neural networks (CNNs) to obtain a better performance based on the mechanism of multiple inputs and multiple output channels of convolutional neural networks, which input multiple modal information at the same time.

Inspired by the work from Zhang et al*.* [12], we propose the CNN-Siam in this paper, which is a novel algorithm based on a convolutional neural network and Siamese network [13, 14] architecture, to leverage information from multimodal data to predict DDI-related events. CNN-Siam regards each drug separately as the input to two CNNs of the Siamese network, where the two CNNs share parameters and learn multimodal information of a drug individually, and then fuse its feature representations and input them into a multilayer perceptron to obtain the prediction output of the DDI event category.

## Results

### Evaluation metrics

**Fig. 1:**
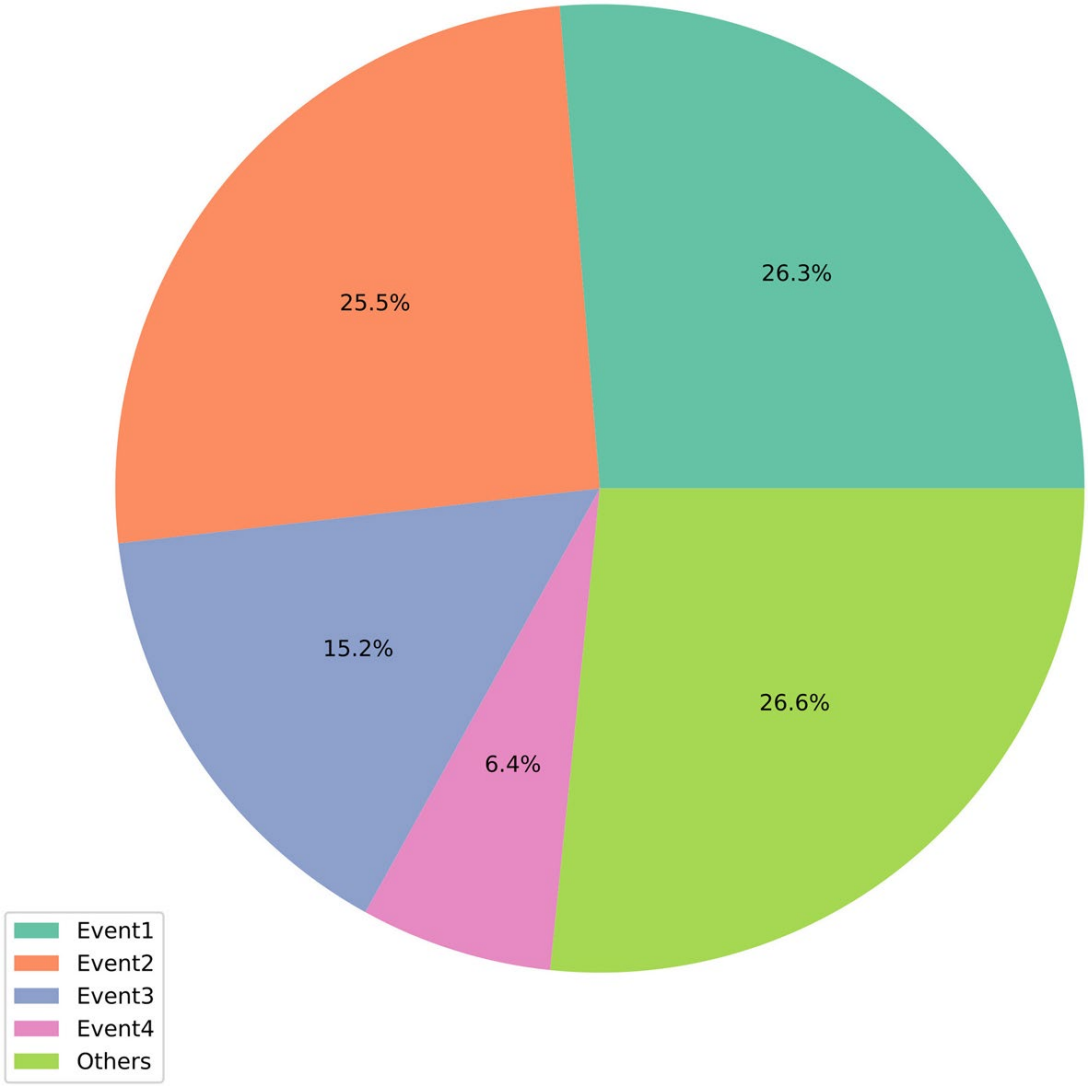
Distribution of the DDI events. Event 1: The metabolism of Drug A is decreased when Drug A is combined with Drug B (19,620 DDIs). Event 2: The risk or severity of the adverse reactions may be increased when Drug A is combined with Drug B (18,992). Event 3: When Drug A is combined with Drug B, the serum concentrations of Drug A may be increased (11,292). Event 4: The serum concentrations of Drug A can decrease when Drug A is combined with Drug B (4772)

Because predicting DDI event categories is a multiclass task, in this paper, we use six metrics to evaluate the effectiveness of the model, namely, the accuracy (ACC), area under the precision-recall curve (AUPR), area under the ROC curve (AUC), precision, recall, and F1-score. AUPR metrics are more suitable for our task than AUC values, where the AUPR scores can better reflect the classification effectiveness of the model in an unbalanced dataset. Because the category imbalance is more significant in our dataset, the common types of DDI events in our dataset are classified into 65 categories. \* MERGEFORMAT Fig. 1 shows the sample situation of each category in our dataset. We use the micro-average approach to compute the AUPR, AUC, precision, recall, and F1-score. The micro-average will aggregate the contributions of all classes to compute the average. In a multiclass classification setup, the micro-average is preferable if there is a class imbalance.

### Model evaluations

In this section, we compare the performance of CNN-Siam with those of several models, including the state-of-the-art method CNN-DDI [12], DDIMDL [6] and DeepDDI [2], followed by some of the more common baseline models: fully connected neural network with 3 layers (DNN), random forest (RF), K-nearest neighbor (KNN) and logistic regression (LR). Since the authors of CNN-DDI did not provide their code, we implemented CNN-DDI with the best strength according to the description of the model in the paper and then ran it on our dataset.

Regarding the setup of the comparison experiments, we used K-fold cross validation training method with K taken as 5, and the scores of the 5 training sessions were averaged as the final scores. About other hyperparameters, the neighbor number of KNN was set to 4, and the number of decision trees of RF was set to 100.

Table 1 shows the comparative results of our model, and we can see that the performance of CNN-Siam is the best thus far (the ACC, AUPR, AUC, and F1 are 0.9237, 0.9627, 0.9986, and 0.9237, respectively), and it substantially outperforms the state-of-the-art method in terms of the accuracy, AUPR, AUC, and F1-score, which are 0.8871, 0.9251, 0.9980, and 0.7496, respectively.

**Table 1:** Results of CNN-Siam and other models

Models	ACC	AUPR	AUC	F1	Precision	Recall
CNN-Siam	**0.9237**	**0.9627**	**0.9986**	**0.9237**	**0.9237**	**0.9237**
CNN-DDI*	0.8681	0.9254	0.9982	0.8681	0.8681	0.8681
CNN-DDI	0.8871	0.9251	0.9980	0.7496	0.8556	0.7220
DDIMDL	0.8852	0.9208	0.9976	0.7585	0.8471	0.7182
DeepDDI	0.8371	0.8899	0.9961	0.6848	0.7275	0.6611
DNN	0.8797	0.9134	0.9963	0.7223	0.8047	0.7027
RF	0.7775	0.8349	0.9956	0.5936	0.7893	0.5161
KNN	0.7214	0.7716	0.9813	0.4831	0.7174	0.4081
LR	0.7920	0.8400	0.9960	0.5948	0.7437	0.5236

A single * indicates the result of our implemented CNN-DDI run on the same dataset. The bold fonts indicate the best results

**Fig. 2:**
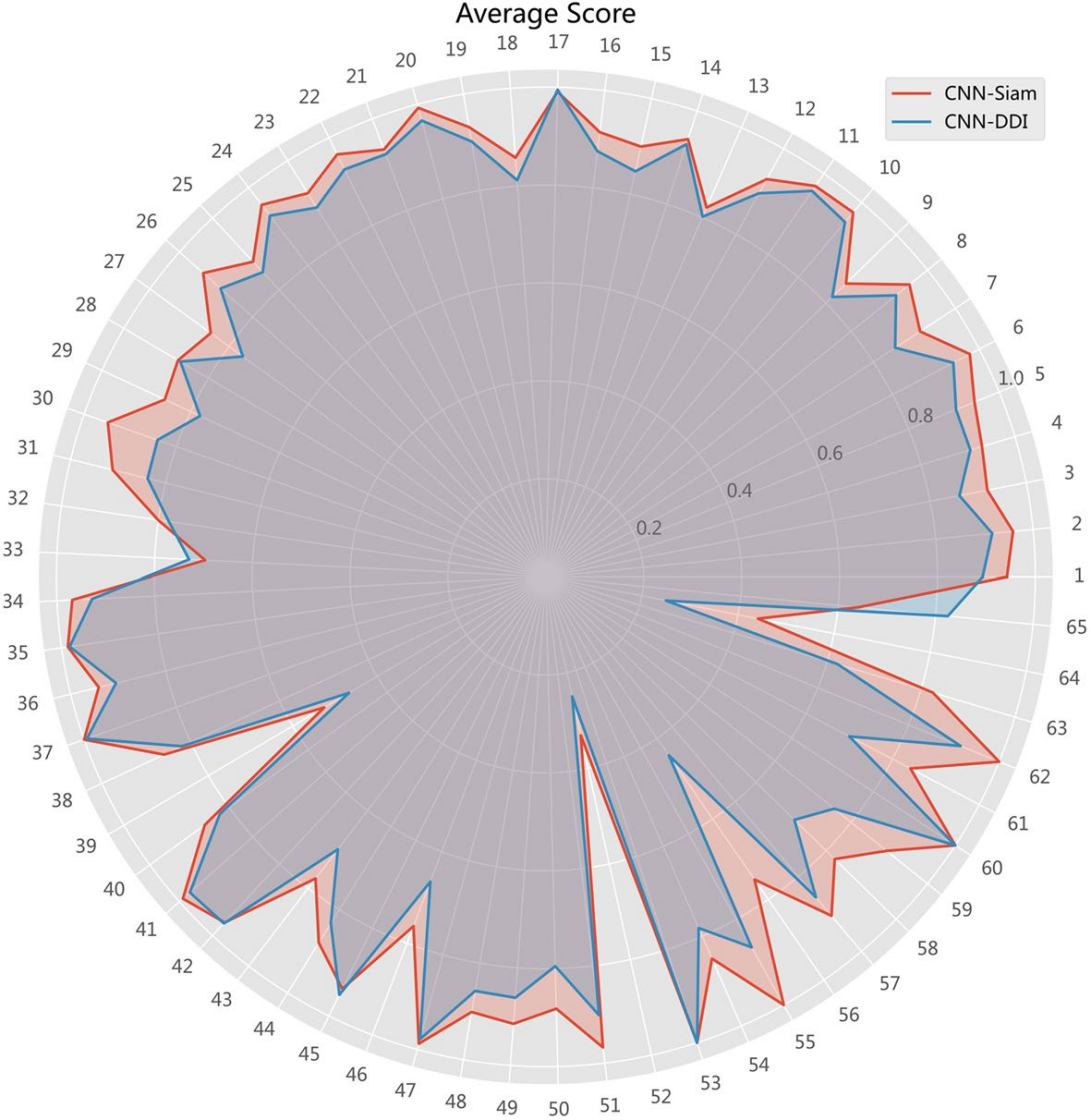
Average scores of *CNN-Siam* and *CNN-DDI* for each DDI event

We compared the combined predictive power of CNN-Siam and CNN-DDI. \* MERGEFORMAT Fig. 2 shows the scores of the models for each of the 65 DDI event types, the scores obtained by averaging 6 metrics by ACC, AUPR, AUC, F1-Score, Precision, and Recall.

### Ablation study

**Fig. 3:**
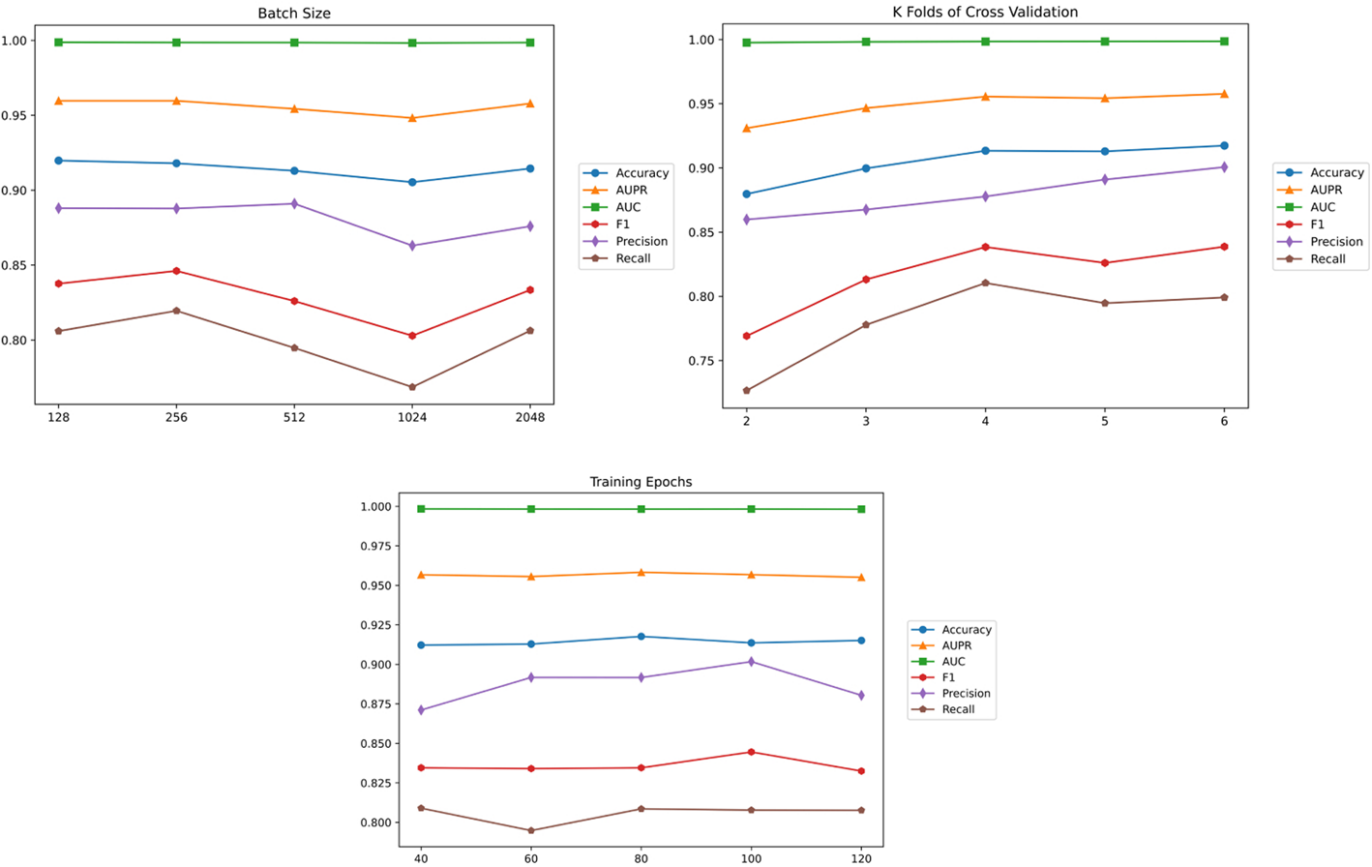
Performance charts with different hyperparameters

We designed corresponding ablation experiments for some technical innovation points in the CNN-Siam algorithm and the overall robustness of the model, and in this subsection, the results of these experiments are presented. (a) For the Siamese network architecture, we experimented with and did not use the twin network architecture. From the findings of recent work [15], we found that summing the input vectors of both drugs and inputting them into the model gives better results when using a single model. Thus, our experiments also aim to compare the results of summing the drug inputs and feeding them into a single CNN model. The results are shown in Table 2, where we can see that CNN-Siam significantly outperforms a single CNN in terms of the accuracy, AUPR, and F1-score. Their values are 0.8879, 0.9425, and 0.8879, respectively, which shows the effectiveness of the Siamese network architecture. (b) CNN-Siam uses the combination of the best optimization algorithm RAdam (rectified Adam) [16] and LookAhead [17], and in the ablation experiments, we compare three cases: using only Adam, using RAdam alone, and using the combination of RAdam and LookAhead; their results are shown in Table 3. It can be demonstrated that RAdam is a better optimizer than Adam and can further improve the performance of the model with the addition of LookAhead. (c) To test the robustness of CNN-Siam, we set up some experiments on hyperparameter tuning, including the batch size, numbers of folds of the cross-validation, and numbers of epochs. The results are shown in \* MERGEFORMAT Fig. 3. From the experimental graphs, we can see that the overall robustness of CNN-Siam is strong, in which more folds may further improve the accuracy of the model in terms of the K-fold cross-validation.

**Table 2:** Results of CNN-Siam and CNN alone

Models	ACC	AUPR	AUC	F1
CNN-Siam	0.9237	0.9627	0.9986	0.9237
CNN-alone	0.8879	0.9425	0.9984	0.8879

**Table 3:** Results of different optimizers

Optimizer	ACC	AUPR	AUC	F1
Adam	0.8213	0.8827	0.9967	0.8213
RAdam	0.9186	0.9583	0.9986	0.9186
RAdam + LA	0.9237	0.9627	0.9986	0.9237

## Conclusion and discussion

Drug molecule interactions play an important part in drug development and the treatment of patients. Since such a large number of drugs cannot be studied based on experimental means, discovering more drug interactions with the help of computational means is currently the best solution. In this work, we proposed a novel algorithm, CNN-Siam, to predict DDI events by fully exploiting the multimodal information of drugs. CNN-Siam learns the representation of a single drug by feeding the chemical substructure, target, and enzyme data of a single drug into two CNNs that share parameters through a Siamese network architecture, and subsequently, the drug pair representations are fused and inputted into a multilayer perceptron for classification. Through experiments, we demonstrate that CNN-Siam, augmented by some advanced optimization algorithms (RAdam and LookAhead), can outperform the best available algorithms and predict the event types of DDIs more accurately.

Among the 65 DDI events predicted in the current dataset, we found that for the first 8 events, the prediction was good, while the scores of the subsequent events were less stable, especially for the event 33 (Drug B may increase the hypertensive activities of drug A), event 39 (the serum concentration of the active metabolites of drug A can be reduced when drug B is used in combination with drug resulting in a loss in efficacy), event 52 (Drug B may increase the hepatotoxic activities of drug A), and event 64 (Drug B may increase the myelosuppressive activities of drug A.). Since the first few DDI events have covered most of the dataset, it is normal to get more satisfactory prediction results, while the later events with poor scores may be more worthy of investigation from other fields. Meanwhile, it can be seen from the experimental results that both CNN-Siam and CNN-DDI have relatively poor prediction results for these events mentioned above, which is a direction we need to focus on for future algorithm improvement.

Overall, the performance of CNN-Siam is good, but it also has some drawbacks. Currently, our model takes a long time to train, so we hope to speed up the training process with some optimizations in future work. We also hope to apply CNN-Siam to larger datasets or add more modal data to further validate its effect on larger datasets and improve its generalization performance in future work. Regarding the architecture of the Siamese network, we can calculate the similarity loss after obtaining the feature vectors of the two drugs, similar to the usual practice of *contrastive learning*. By adding the similarity loss and classification loss together, the model can more clearly learn and more accurately predict the DDI similar to the known DDI.

## Method

### Dataset

In this study, we used a public dataset collected by Deng et al. [6] from the DrugBank online database. This dataset contains multiple pieces of information on 572 drugs, including the chemical structures, targets, enzymes, and pathways. There are 74,528 paired DDIs, and the number of known DDIs is 37,264. They counted these 37,264 known DDIs and finally filtered 65 more common DDI types. Top 4 frequent events are: (1) the metabolism of drug A can be decreased when combined with drug B (19,620); (2) the risk or severity of adverse effects can be increased when drug A is combined with drug B (18,992); (3) the serum concentration of drug A can be increased when it is combined with drug B (11,292). (4) the serum concentration of drug A can be decreased when it is combined with drug B (4772). Details of all events are provided in supplementary-data of Deng et al. [6].

From their experiments, we can see that the prediction accuracies of the DDIs are higher when three modal information of the chemical structures, targets, and pathways are selected. Thus, we only use these three feature data of drugs.

In training the model, we used two techniques for the data separately: K-fold cross validation and Mixup [18]. K-Fold cross validation is a common cross validation method that divides the dataset into $K$ parts, and for each training loop, $K-1$ parts of the data are used for training, and the remaining part is used as the validation set, the training will take $K$ loops. In our experiments, we divide the dataset into 5 parts and use 4 parts of the data for each training loop, and the remaining part of the data is used as the validation set. During the training process, we recorded the results of each training session and finally took the average value as the final result. *Mixup* is a data argumentation method that increases the size of the dataset by performing some transformations on the data to improve the generalization ability of the model, and it is calculated as:

1 $$\lambda =Beta\left(\alpha ,\beta \right)$$

$$MixedBatch=\lambda \times batc{h}_{x1}+\left(1-\lambda \right)\times batc{h}_{x2}$$

where $batch\_x1$ is a subset randomly sampled from the dataset, and $batch\_x2$ is another subset randomly sampled again after disrupting the dataset. $\lambda$ is a randomly generated number that obeys a beta distribution and is controlled by the two hyperparameters $\alpha$ and $\beta$. In our experiments, we set both $\alpha$ and $\beta$ to 0.5 to ensure that $\lambda$ takes a value of approximately 0.5, thus ensuring the equilibrium of the data.

### CNN-Siam algorithm

#### Drug data preprocessing

The extraction of the drug features and their conversion to a specific representation is the most important step in the construction of a model. Based on previous work [6], we chose to use one-hot encoding to transform the three modal data of a drug, i.e., chemical structure, target, and enzyme. One-hot encoding is a commonly used encoding method that transforms the chemical structure of each drug into a vector. Each element in the vector takes the value 0 or 1, where 0 means no atom at a position and 1 indicates the presence of atoms at that position. However, the dimensionality of the drug feature vector after one-hot encoding is too high, and the sparsity is high (most values are 0). Thus, we use the Jaccard similarity to calculate the similarity between two drugs, and the formula for the Jaccard similarity is:

2 $$Jaccard=\frac{\left|A\cap B\right|}{\left|A\cup B\right|}=\frac{\left|A\cap B\right|}{\left|A\right|+\left|B\right|-\left|A\cap B\right|}$$

where $A$ and $B$ represent the one-hot feature vector of the drugs, $\left|A\cap B\right|$ represents the intersection of Drug A and Drug B, and $\left|A\cup B\right|$ represents the union. The Jaccard similarity is in the range of [0, 1], and the larger the value is, the higher the similarity of the two drugs. After calculating the Jaccard similarity, we convert the drug feature vector into 572 dimensions; thus, the input of the model is 2 * 572 * 3 dimensions.

#### Model description

**Fig. 4:**
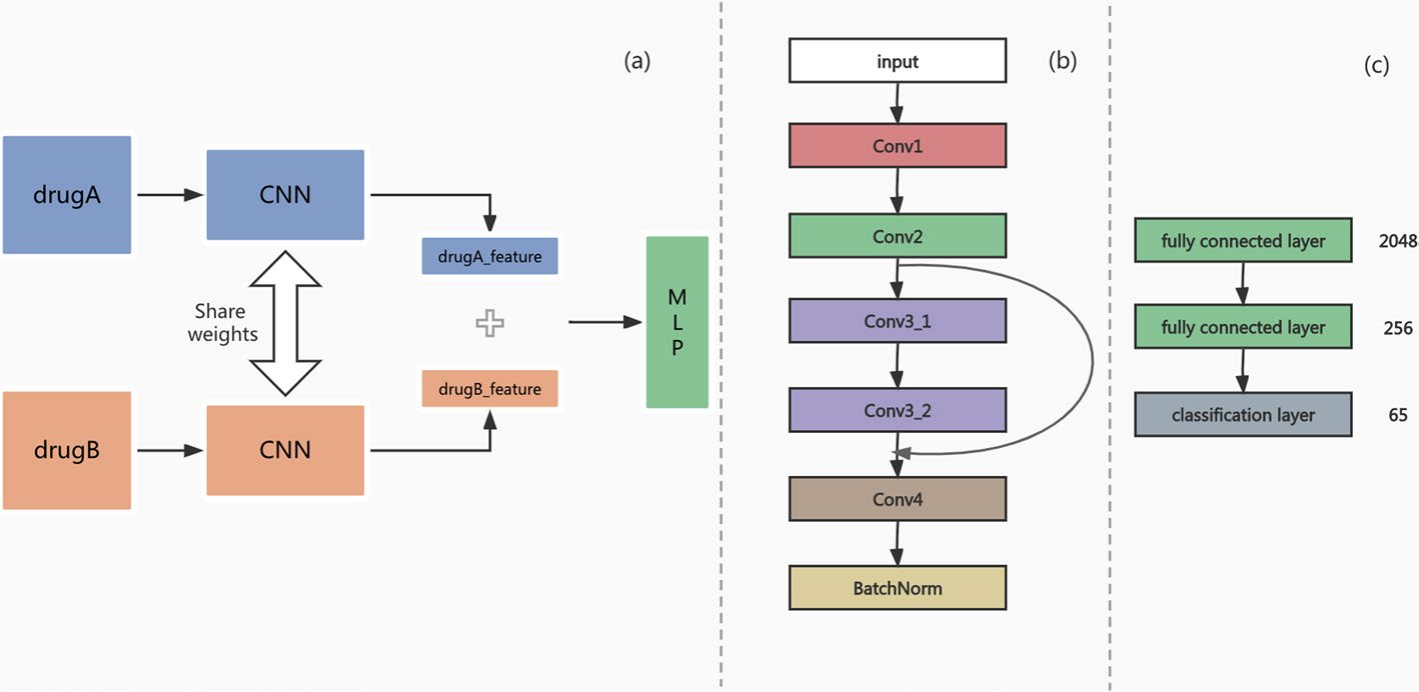
The framework of CNN-Siam. **a** Workflow: Inputting Drug A and Drug B into two weight-sharing CNNs to obtain the feature outputs, summing them, and finally inputting them into a multilayer perceptron for classification. **b** Model architecture of the CNN. It consists of 5 convolutional layers and 1 normalization layer, where the output of the 2nd convolutional layer is added to the input of the 5th convolutional layer to achieve a residual connection. **c** Architecture of the MLP. Two hidden layers and one classification layer

The main framework of the model is shown in \* MERGEFORMAT Fig. 4a. The input of the model is the feature vectors of two drugs, *Drug A* and *Drug B*. Moreover, the dimensions of *Drug A* and *Drug B* are 572 * 3, and the two feature vectors *drugA_feature* and *drugB_feature* are obtained after the computation of a CNN, respectively. In the experiments of Lin et al. [15], the authors found that by simply adding the two drug feature vectors and then inputting them into the model gives better results. Thus, we tested the results of concatenating *drugA_feature* and *drugB_feature* in the dimensions and adding them directly and proved that the direct addition is better. We have also done experiments to verify this, and the data shows that concatenating gives 0.78 ACC, 0.92 AUPR, 0.97 AUC and 0.79 F1-Score, while directly adding them together gives a better result of 0.92 ACC, 0.96 AUPR, 0.99 AUC and 0.92 F1-Score. Therefore, the feature vectors are summed and inputted into a multilayer perceptron, which consists of two hidden layers and one classification layer. The number of neurons in each layer is 2048 and 256, the activation function is ReLU, and the output results correspond to the classification probabilities of 65 DDIs. (\* MERGEFORMAT Fig. 4c).

*Convolutional neural network* CNNs have been widely used in the field of deep learning for computer vision and have achieved satisfactory results. Because of the development of deep learning techniques, an increasing number of studies have started to replace MLPs with CNNs as backbone networks to solve problems in the life sciences. The benefit of CNNs is that they can fully extract local as well as global information. In addition, they can share parameters and save considerable computational overhead compared to MLPs. In this paper, for each single CNN of CNN-Siam, the structure is shown in Fig. 4b. Based on the CNN-DDI [12], we keep only five convolutional layers, each with 64, 128, 128, 128, and 256 convolutional kernels of size 3*1. After the last convolutional layer, we add a batch normalization layer to prevent the distribution shift of the feature vectors computed and summed by the CNNs and to reduce a certain degree of overfitting.

In addition, we also implemented a residual connection [19], which adds the output of the *conv2* convolutional layer directly to the output of the *conv3_2* convolutional layer. This process has the advantage of enabling the subsequent convolutional layers to learn the features better and reduces the problem of gradient disappearances, allowing even deeper networks to be trained successfully and increasing the training speed of the model. The residual connection is calculated as:

3 $$y=f\left(x\right)+x=\sigma \left[{W}_{2}\left[\sigma \left({W}_{1}x+{b}_{1}\right)\right]+{b}_{2}\right]+x$$

where $x$ is the input, $y$ is the output, $f\left(x\right)$ is the output of the convolutional layer, ${W}_{1}$ and ${W}_{2}$ are the weights of the convolutional layers, ${b}_{1}$ and ${b}_{2}$ are the biases, and $\sigma$ is the activation function.

*Siamese network* The Siamese network is a special kind of neural network that consists of two identical networks that completely share the parameters. Usually, the twin network is used to calculate the similarity of the two inputs, and the two feature vectors obtained from the calculation are subjected to similarity losses. Then, the parameters are updated through backpropagation. In our model, the two drugs are input into two identical CNNs so that the two CNNs can simultaneously learn some features that are similar between the two drugs. Moreover, these features can help the model to identify other drug pairs to determine whether there is a DDI for these drug pairs. The twin network is insensitive to the order of the drugs, i.e., the model can learn similar features between two drugs without affecting the learning of individual drug features due to the change in the input order.

*Loss function* On the choice of the loss function, we use the Focal Loss [20], and the equation is shown below:

4 $$FL\left({p}_{t}\right)=-{\left(1-{p}_{t}\right)}^{\gamma }log\left({p}_{t}\right)$$

where ${p}_{t}$ is the probability of the prediction for Category t; $\gamma$ is the hyperparameter, when *γ* = 0; FL is the cross-entropy loss function; and when *γ* = 2, FL is the Focal Loss. We set γ to 2. Regarding the probability of Classification ${p}_{t}$, the larger ${p}_{t}$ is, i.e., the more accurate the classification, the smaller the value of the FL loss. Moreover, the smaller ${p}_{t}$ is, the larger the value of FL. This is equivalent to the more inaccurately classified categories. The larger value of the loss function will be given. This causes the model to be more focused on those misclassified samples (i.e., those categories with a particularly small number of samples), thus allowing the model to better learn the features of these samples. Furthermore, the classification accuracy of the model is improved. For our dataset, the first three categories account for almost 70% of the samples, so the Focal Loss is a necessary choice.

*Optim Algorithms* RAdam (Rectified Adam) [16], a variant of the classical optimizer Adam, LookAhead [17], is a novel algorithm that can assist the optimizer in parameter updating. Regarding a conventional training process of a deep learning model, the model is first defined (architecture is determined), and then the parameters of the model are randomly initialized. After entering the training step, the data are divided into multiple mini-batches. Then, one mini-batch is input at a time to calculate the prediction result. The loss function is used to compare the prediction with the real result to obtain the loss value, the loss gradient is passed back, and the optimizer is used to update the model parameters. Therefore, the choice of the optimizer is a crucial part of the model. The improvement of RAdam over Adam is that it dynamically turns on or off the adaptive learning rate according to the dispersion of the variance at the early stage of training, which makes the model less prone to fall into the local optimal solution. Moreover, it has the advantage of a fast convergence of vanilla Adam, which is equivalent to providing a combination of Adam. The LookAhead optimization algorithm is an auxiliary algorithm of the optimizer, which is based on the principle of maintaining two sets of model weights internally. One set of weights is responsible for exploring fast updates forward, and the other set of weights is updated slowly. However, this set can provide long-term stability. Furthermore, these two sets of parameters can be interpolated, thus improving the training stability and convergence speed. The greatest advantage is that there is no need for manual hyperparameter tuning. Overall, the combination of RAdam and LookAhead is a very good optimization algorithm that can ensure stable training of the model and reduce the computational and time costs of manual hyperparameter adjustment.

## Data Availability

The datasets generated and/or analysed during the current study are available in the Github repository, https://github.com/Parsonlee/DDI
